# Waist circumference measures: cutoff analyses to detect obesity and cardiometabolic risk factors in a Southeast Brazilian middle-aged men population - a cross-sectional study

**DOI:** 10.1186/1476-511X-13-141

**Published:** 2014-09-01

**Authors:** Alessandro de Oliveira, Paula G Cocate, Helen Hermana M Hermsdorff, Josefina Bressan, Mateus Freitas de Silva, Joel Alves Rodrigues, Antônio José Natali

**Affiliations:** Department of Nutrition and Health, Universidade Federal de Viçosa, Av. PH Rolfs, s/n, Viçosa, Minas Gerais 36570-000 Brazil; Department of Physical Education Science and Health, Universidade Federal de São João del-Rei, São João del-Rei, Minas Gerais Brazil; Department of Physical Education, Universidade Federal de Viçosa, Viçosa, Minas Gerais Brazil

**Keywords:** Obesity, Waist circumference, Non-communicable disease, Metabolic syndrome

## Abstract

**Background:**

Low-cost practical and reliable tools to evaluated obesity-related cardiometabolic diseases are of clinical practice and public heath relevance worldwide. The aims of this cross-sectional study were to determine the anatomical point of waist circumference that best identify overweight, obesity and central obesity in Southeast Brazilian middle-aged men and to test the relationships of its cutoff points with metabolic syndrome (MetS), insulin resistance (IR) and cardiometabolic risk factors.

**Methods:**

Three hundred men [age: 51 (47–54)] underwent anthropometric, body composition, clinical, sociodemographic and blood plasma biochemical evaluations.

**Results:**

The umbilical line circumference (WC_UL_) was the best predictor for overweight (total body fat ≥ 20%; cutoff point: 88.8 cm), obesity (total body fat ≥ 25%; cutoff point: 93.4 cm) and central obesity (abdominal area fat ≥ 34.6%; cutoff point: 95.6 cm) as measured by dual beam X-ray absorptiometry. Subjects with WC_UL_ ≥ 88.8 cm or ≥ 93.4 cm showed significantly higher values for MetS, IR and cardiometabolic risk factors (i.e. glucose and lipid profiles, blood pressure). The occurrence of WC_UL_ ≥ 88.8 cm was positively associated (p <0.01) with the prevalence of MetS and cardiometabolic risk factors and increased the central obesity prevalence by 19.3% while that of WC_UL_ ≥ 93.4 cm was associated with the prevalence of MetS, IR and cardiometabolic risk factors.

**Conclusions:**

WC_UL_ measure seems to be the best predictor for overweight, obesity and central obesity in urban residents Southeast Brazilian middle-aged men; and the WC_UL_ cutoff point (88.8 cm) is significantly associated with MetS, IR and cardiometabolic risk factors in the studied population.

## Introduction

Obesity is a major public health problem worldwide. The accumulation of fat in the body, especially in the central region, is positively associated with the development of chronic non-communicable diseases (NCDs) [1–4].

Metabolic syndrome (MetS) is characterized by an aggregation of relevant cardiometabolic risk factors, such as abdominal obesity, dyslipidaemia, high blood pressure and high fasting blood glucose [3]. Metabolic syndrome is more prevalent in Brazilian subjects over 40 years of age, with prevalence ranging from 23% up to 39.2%, depending on the geographic region, gender, age and criteria of diagnosis [5, 6]. Insulin resistance (IR) is a common pathologic state in which target cells fail to respond to ordinary levels of circulating insulin and, like MetS, it is an important metabolic risk factor for diabetes and cardiovascular diseases [7]. However, no data on IR prevalence in the Brazilian population is available and IR is not internationally agreed upon as a criterion in the diagnosis of MetS, making it a matter of debate [8].

The early diagnosìs of MetS, IR and related cardiometabolic risk factors in populations is of clinical importance and economical relevance. Nevertheless, the diagnosis of both MetS and IR in large-scale populations is limited by the high cost of biochemical analyses and the exposure of the volunteer to invasive procedures (i.e. discomfort to locate the anatomical points for anthropometric measurements; and blood collection). Thus, low-cost practical and reliable measurements such as central circumferences are of clinical and public health relevance worldwide. The waist circumference (WC) measure has been shown as a good predictor for central obesity [2, 9] and hence cardiometabolic risk factors [10], IR [11] and MetS prevalence [12]. However, there are limitations in the use of such measure (i.e. midpoint between the superior border of the iliac crest and inferior margin of the rib), mainly in overweight and obese subjects, as some anatomical points are not precisely identified on them [13, 14]. Moreover, the lack of a standardized method for WC measures by the organizations responsible for NCDs standards hinders comparison between different geographic regions [15, 16].

Along with early diagnosis of NCDs by using simple measurements, it is important to set cutoff points for specific populations as WC differs among different ethnic groups due to distinct prevalence of cardiometabolic risks [17]. Previous studies have suggested different values of WC cutoff points for the diagnosis of obesity and chronic diseases as compared to those generally used (i.e. Alberti et al. [3]: 90 cm for South American population) [18, 19]. For example, 94 cm for 21–95 old Latin Americans (Mexico, El Salvador, Venezuela, Colombia and Paraguay) [18] men as well as 90.2 cm [19] and 88 cm [20] for urban residents in the northeast region of the Brazil aged 20–59 years were suggested. However, studies suggesting WC cutoff points for the early detection of NCDs, specifically in Brazilian middle-aged men (40–59 years old), are not found.

Therefore, this study was carried out to: (a) determine one anatomical point of WC that best identify overweight, obesity and central obesity in urban resident Southeastern Brazilian middle-aged men; and (b) test the relationships of its cutoff points with MetS and IR prevalence and, cardiometabolic risk factors in this population.

## Methods

### Study population

This cross-sectional study was carried out between March and December 2011. By convenience, we studied the population of middle-aged men who were staff members of the Federal University of Viçosa, located in the Brazilian southeastern city of Viçosa, Minas Gerais state. This population consisted of 1,774 (N) men aged between 40–59 years. The sample size was calculated using the confidence level of 95% and the prevalence for obesity in Brazilian men (i.e. 17.5%), as detected by the Brazilian Health Minister [4], and 4% of sampling error, which resulted in 291 (n) participants as a minimum sample size required. The Epi Info software, version 6.04, for cross-sectional studies [21] was used to estimate sample size.

To select the subjects of this study all 1,774 staff members were listed and numbered alphabetically and those numbered multiple of 6 (N/n: 1,774/291) were chosen. In the event of meeting the exclusion criteria the subject was replaced by his predecessor in the list. Eight hundred fifty-six subjects were interviewed and 300 of them were eligible to take part in the present study.

This study excluded subjects who self-declared: body weight alterations of ≥ 3 kg, altered levels of physical activity and eating habits in the three months preceding the study; thyroid diseases, heart failure, cerebrovascular diseases, infectious and/or inflammatory diseases, diseases of the gastrointestinal tract, liver and chronic kidney and/or history of kidney stones, cancer in the previous ten years, eating disorders (anorexia and bulimia) and food allergies. Subjects using diuretics or drugs that could alter food intake and/or metabolism of nutrients were also excluded. Pacemaker and/or prosthetic users were excluded as it could affect the DXA result analyses. Elite athletes were excluded as they could exhibit an inflammatory condition due to exercise training stress.

This study is in accordance with the resolution 196/96 from the Brazilian Ministry of Health regarding research involving human subjects and was approved by the Ethics Committee on Human Research of the Federal University of Viçosa (Of. Ref. n° 069/2010/CEPH). Only participants who signed the consent form in accordance with the Declaration of Helsinki were selected.

### Anthropometry and body composition measurements

Anthropometry and body composition measurements were carried out after a 12-hour fast and the subjects were instructed to perform no physical activities of moderate and high intensity and no caffeine and alcohol ingestion in the 48 hours prior to the test.

Body weight and height were determined following the protocol described by Gordon et al. [22], using a digital scale with stadiometer (2096PP, Toledo, São Bernardo do Campo, SP, Brazil). Body mass index (BMI) was calculated using the equation proposed by Quetelet and the subjects were categorized as: eutrophic (18.5 to 24.9 kg/m^2^), overweight (25.0 to 29.9 kg/m^2^) or obese (≥30 kg/m^2^), according to the criteria set by the World Health Organization [23].

Waist circumferences were measured on three anatomical points: (a) narrowest waist (WC_NR_) [24] (i.e. nearly 1 cm below the last rib); (b) midpoint between the superior border of the iliac crest and inferior margin of the rib (WC_MD_) [2, 3] (i.e. nearly 3 cm above the umbilical line); and (c) at the umbilical line (WC_UL_) [25]. Waist circumferences were measured in triplicate using a flexible, no stretching tape (TR4010, Sanny, São Bernardo do Campo, SP, Brazil) and the average value for each anatomical point was considered for data analyses.

Total body scan was performed by dual beam X-ray absorptiometry (DXA) (LUNAR, GE, Encore software version 13:31, Madison, WI, USA) to determine the percentages of total body fat (%BF) and abdominal area fat (%AAF). Abdominal area fat is the body fat detected in the area between the superior border of the iliac crest and the inferior border of the last rib. Overweight and obesity cutoff values were set at 20% and 25% of %BF [26, 27], respectively. Since there is no cutoff points for %AAF reported, the percentage found in the 75th percentile of %AAF in the present sample was used for central obesity.

### Blood pressure, blood glucose, insulin and serum lipid profile measurements

Systolic (SBP) and diastolic blood (DBP) pressure were measured using an automatic inflation blood pressure monitor (BP3AA1-1, G-Tech, OnboElectronicCo, Schenzen, China), registered at ANVISA (No. 80275310004), following the VI Brazilian Guidelines on Hypertension [28].

Blood samples were collected from the antecubital vein and the serum was separated by centrifugation at 2.225 g for 15 min at room temperature (Sigma 2–3, Sigma Laborzentrifuzen, OsterodeamHarz, Germany). Blood glucose was measured using the glucose oxidase method (Cobas Mira Plus, Roche Diagnostics, GmbH, Montclair, NJ, USA), and insulin was measured by electrochemiluminescence (Modular Analytics, E170, Roche Diagnostics, GmbH, Mannheim, Germany).

Serum total cholesterol, high-density lipoprotein (HDL-C) and triglycerides were determined by an enzymatic colorimetric method (Cobas Mira Plus, Roche Diagnostics GmbH, Montclair, NJ, USA). The atherogenic index was calculated as the total cholesterol to HDL-C ratio [29].

### Determination of metabolic syndrome, insulin resistance and cardiometabolic risk factors

The MetS was considered prevalent in subjects who exhibited three or more factors related to waist circumference (WC_MD_ ≥ 90 cm), hyperglycaemia (glucose ≥ 100 mg/dL), dyslipidaemia (HDL-C < 40 mg/dL), hypertriglyceridemia (≥150 mg/dL) and/or high blood pressure (SBP ≥ 130 mmHg or DBP ≥ 85 mmHg), according to the criteria and cutoff points suggested by Alberti et al. [3].

The homeostasis model assessment (HOMA-IR) was used to estimate IR by using the equation proposed by Matthews et al. [30]. The cutoff value used for the IR diagnosis was 2.7 as suggested by Geloneze et al. [31].

The following values were set as cardiometabolic risk factors [32, 33]: triglycerides ≥ 150 mg/dl (hypertriglyceridemia); total cholesterol ≥ 200 mg/dl and HDL-C < 40 mg/dl (dyslipidaemia) and glucose ≥ 99 mg/dl (hyperglycaemia). The participants were classified as hypertensive when systolic and diastolic blood pressures were ≥ 140 and ≥ 90 mmHg, respectively, [28] and it was considered a cardiometabolic risk when atherogenic index was ≥ 5 [29].

### Lifestyle

The subjects who participated in this study occupied working positions classified as levels A, B, C, D, and E, or professor. To evaluate how lifestyle and occupation influenced the level of physical activity they were grouped according to their education level and working positions: Group ABC was composed of technical and administrative staff members, classified as A, B and C, with an education level up to high school. Group DEProf was composed of technical and administrative staff members levels D and E and professors, all college-educated.

The participants were asked about their current smoking status (yes/no) and alcohol consumption (types of alcoholic beverages consumed - beer, wine and/or spirits, frequency and weekly quantity in mL). High alcohol consumption was defined as a weekly intake over 21 units [34].

The full version of the International Physical Activity Questionnaire [35] was applied and subjects were categorized as sedentary/moderately active or active/very active.

### Statistical Analysis

Data normality was assessed by the “Smirnov-Kolmogorov” test. For data exposure, we used descriptive statistics composed by mean values and standard deviation or median and interquartile range for continuous variables and frequency for categorical variables. After logarithmic transformation the WC_NR_, WC_MD_ and WC_UL_ values were compared by ANOVA one way followed by the post hoc Tukey test. The physical activity levels were compared using Chi-Square. The Student’s t test was used for independent samples, or its nonparametric equivalent, the Mann-Whitney test, to confirm the existence of differences between mean values per group.

The receiver operating characteristic curve (ROC) was used to detect the best circumference cutoff, sensitivity (Sens) and specificity (Spec) in relation to the cutoff points: 21%BF and 25%BF and 34.6%AAF. The areas under the curve and confidence intervals of 95% (95% CI) were also determined. The univariate and multivariate regression analysis according to Poisson was used to estimate the prevalence ratio (95% CI) of subjects with hyperglycaemia, dyslipidaemia, hypertensive and MetS (dependent variables). In these analyses the WC cutoff point (88.8 cm) served as independent variable and the lifestyle factors as covariates.

Data processing and analysis were carried out with the software SPSS version 16.0 (SPSS Inc. Chicago, IL, USA) and STATA 9.1. The value used for all variables and two-tailed analyses was p # 0.05.

## Results

The general characteristics of the participants are shown in Table 1. There was a high incidence of technical administrative staff members, moderate prevalence of high alcohol consumption (20.3%) and low prevalence of smoking (12.7%). In addition, most participants were self-declared as physically active or very active (69.4%). The BMI assessment showed overweight and obesity in 42.6% and 12.3% of subjects, respectively. The following prevalence percentages were observed: 61.7% for dyslipidaemia, 30.7% for hypertriglyceridemia, 37.7% for high atherogenic index, 34.3% for hypertension, 18.3% for hyperglycaemia and 9% for IR. There was a prevalence of 28.3% for MetS, according to the criteria suggested by Alberti et al. [3]. After comparing the measured waist circumferences (WC_NR_, WC_MD_, WC_UL_), we observed no statistical difference between WC_NR_ and WC_MD_. However, WC_UL_ was higher than WC_NR_ and WC_MD_.

**Table 1:** **General characteristics of the studied subjects**

Age (y)	51 (47–54)^a^
ABC work position (number/%)^b^	199/66.3
High alcohol consumption (number/%)	61/20.3
Smokers (number/%)	38/12.7
Physical activity levels (number/%)	
Sedentary or moderately active	92/30.6
Active or very active	208/69.4^#^
Body mass index (kg/m^2^)	25.8 ± 3.43
Total body fat (%)	22.76 ± 7.14
Abdominal area fat (%)	26.34 ± 10.52
WC_NR_ (cm)	88.7 ± 8.4
WC_MD_ (cm)	90.4 ± 9.5
WC_UL_ (cm)	92.2 ± 9.3^*^
Systolic blood pressure (mmHg)	126 ± 14
Diastolic blood pressure (mmHg)	81 ± 10
Glucose (mg/dl)	89 (83–95)
Insulin (μIU/mL)	5.2 (3.4-8.1)
HOMA-IR	1.14 (0.73-1.81)
Total cholesterol (mg/dl)	214.8 ± 40.9
HDL-C (mg/dl)	44 (38–53)
Triglycerides (mg/dl)	116.5 (82.5-166)
Atherogenic index	4.68 (3.82-5.60)

n = 300; WC_NR_, waist circumference at the narrowest waist, WC_MD_, waist circumference at the midpoint between the superior border of the iliac crest and the inferior margin of the rib, WC_UL_, waist circumference at the umbilical line. HOMA-IR, insulin resistance index; HDL-C, high density lipoprotein-cholesterol.

^a^Data are mean ± SD or median and interquartile range of 300 subjects, according to normal distribution of the variables.

^b^ABC: technical staff at A, B and C work positions.

^*^Statistically different from WC_NR_ and WC_MD_.

^**#**^Statistically different from Sedentary or moderately active.

The waist circumference values measured at the three different anatomical points showed strong and significant associations (p ≤ 0.01) with overweight, obesity and central obesity (Table 2) as determined by DXA. Interestingly, the WC_UL_ was observed as the best measure to identify the prevalence of overweight and obesity, according to the percentages of total body fat (≥20% and ≥ 25%, respectively) and of central obesity (abdominal area fat ≥ 34.6%). The WC_UL_ best cutoff values for overweight, obesity and central obesity detection as regard to the smallest difference between sensitivity and specificity were 88.8 cm, 93.4 cm and 95.6 cm, respectively.

**Table 2:** **AUC (95**% **CI) for overweight, obesity and central obesity and different anatomical points of central circumference in middle-aged men**

	Overweight (>20% BF)	Obesity (>25% BF)	Central obesity (34.6% AAF)
WC_NR_			
AUC	0.877 (0.839-0.915)	0.905 (0.870-0.940)	0.898 (0.861-0.936)
Cutoff (cm)	86.4	89.4	91.6
Sensitivity (%)	77.6	84.6	80.3
Specificity (%)	77.9	83.6	80.4
WC_MD_			
AUC	0.909 (0.877-0.942)	0.919 (0.890-0.948)	0.897 (0.861-0.933)
Cutoff (cm)	87.3	91.4	93.8
Sensitivity (%)	82.1	83.8	80.3
Specificity (%)	82.7	82.0	79.9
WC_UL_			
AUC	0.925 (0.893-0.953)	0.923 (0.894-0.952)	0.902 (0.861-0.942)
Cutoff (cm)	88.8	93.4	95.6
Sensitivity (%)	82.5	84.6	81.6
Specificity (%)	83.0	84.7	81.2

AUC, area under curve. CI, confidence intervals. WC_UL_, waist circumference at the umbilical line. WC_MD_, waist circumference at the midpoint between the superior border of the iliac crest and the inferior margin of the rib. WC_NR_, waist circumference at the narrowest waist.

We used the two smaller WC_UL_ cutoff points (88.8 cm and 93.4 cm) to test its relationships with Mets, IR and cardiometabolic risk factors (Table 3). The occurrence of WC_UL_ ≥ 88.8 cm or 93.4 was positively associated (p<0.01) with the prevalence of MetS and the cardiometabolic risk factors studied.

**Table 3:** **Cardiometabolic risk factors in relation with cutoff of points for waist circumference measured at the umbilical line**

	Cutoff WC _UL_^a^		p-value ^b^	Cutoff WC _UL_^a^		p-value ^b^
	< 88.8 cm (n = 120)	≥ 88.8 cm (n = 180)		< 93.4 cm (n = 173)	≥ 93.4 cm (n = 127)	
Glucose (mg/dl)	85 (81–93)^c^	90 (85–97)	< 0.001	87 (82–93)	90 (85–97)	< 0.001
HOMA-IR	0.74 (0.52-1.05)	1.58 (1.09-2.22)	< 0.001	0.84 (0.61-1.17)	1.76 (1.23-2.49)	< 0.001
Total cholesterol (mg/dl)	206.1 ± 38.0	220.0 ± 41.9	0.004	206.1 ± 36.4	225.7 ± 44.0	< 0.001
HDL-C (mg/dl)	48 (41–57)	43 (37–50)	< 0.001	46 (40–55)	42 (37–50)	0.004
Triglycerides (mg/dl)	90 (70–127)	136 (101–208)	< 0.001	99 (72–139)	143 (107–246)	< 0.001
Atherogenic index	4.15 (3.46-4.97)	4.93 (4.27-5.92)	< 0.001	4.37 (3.59-5.10)	5.05 (4.30-6.42)	< 0.001
Triglycerides/HDL-C	1.85 (1.36-2.74)	3.31 (2.11-4.90)	< 0.001	2.06 (1.52-3.31)	3.56 (2.35-5.79)	< 0.001
Systolic blood pressure (mmHg)	122.1 ± 13.1	128.7 ± 13.6	< 0.001	129.3 ± 13.6	123.8 ± 13.5	0.001
Diastolic blood pressure (mmHg)	77.5 ± 9.0	83.4 ± 9.5	< 0.001	78.8 ± 9.2	84.1 ± 9.6	< 0.001

WC_UL_: Waist circumference at the umbilical line; HDL-C: high density lipoprotein cholesterol; HOMA-IR: homeostatic model assessment insulin resistance; n: number of subjects.

^a^percentage of body fat cutoff points suggested as for the best sensitivity and specificity relation (see Table 2).

^b^p-values from Student t-test or Mann–Whitney test.

^c^Data are mean ± SD or median and interquartile range.

We then tested the associations of the prevalence of MetS, IR and cardiometabolic risk factors with the suggested cutoff points for WC_UL_ (Table 4). Significant associations of this circumference values for overweight (i.e. > 88.8 cm) with the diagnosis of hypertension, dyslipidaemia, hypertriglyceridemia and lower HDL-C, independently of confounding variables (i.e. smoking and frequency of alcoholic beverage consumption) were observed. All subjects diagnosed with insulin resistance presented WC_UL_ higher than 88.8 cm, which made the statistical calculation impossible. Moreover, using WC_UL_ higher than 88.8 cm we observed the increase of 19.3% in central obesity prevalence as compared to WC_MD_ ≥ 90 cm (data not shown). Finally, when we tested the WC_UL_ cutoff point for obesity (i.e. > 93.4 cm) significant associations with MetS, IR and cardiometabolic risk factors were found.

**Table 4:** **Prevalence ratio of MetS, and cardiometabolic risk factors in relation with cutoff points for WC**_**UL**_ **calculated using a univariate and multivariate regression analysis according to Poisson**

	≥ 88.8 cm WC _UL_^a^	≥93.4 cm WC _UL_^a^
Non adjusted^b^		
High fast glucose^d^	1.778 (1.028-3.073)^*^	1.519 (0.942-2.450)
Insulin resistance^e^	^***^	7.833 (2.773-22.128)^**^
High total cholesterol^f^	1.174 (0.967-1.422)	1.291 (1.083-1.538)^**^
Low HDL-C^g^	1.316 (1.126-1.538)^**^	1.295 (1.085-1.545)^**^
High triglycerides^h^	2.561 (1.634-4.015)^**^	2.554 (1.776-3.673)^**^
Atherogenic index^i^	2.123 (1.472-3.062)^**^	1.844 (1.374-2.477)^**^
High systolic blood pressure^j^	1.548 (1.088-2.203)^*^	1.501 (1.099-2.051)^*^
High diastolic blood pressure^k^	2.000 (1.354-2.954)^**^	1.881 (1.359-2.603)^**^
Metabolic Syndrome^l^	3.917 (1.917-8.001) ^**^	4.401 (2.466-7.855) ^**^
After Adjustment^c^		
High fast glucose^d^	1.936 (1.129-3.318)^*^	1.632 (1.018-2.615)^*^
Insulin resistance^e^	^***^	6.829 (2.414-19.317)^**^
High total cholesterol^f^	1.154 (0.952-1.400)	1.271 (1.066-1.515)^**^
Low HDL-C^g^	1.361 (1.165-1.591)^**^	1.327 (1.113-1.582)^**^
High tryglicerides^h^	2.516 (1.604-3.946)^**^	2.500 (1.730-3.611)^**^
Atherogenic index^i^	1.896 (1.418-2.534)^**^	2.217 (1.543-3.186)^**^
High systolic blood pressure^j^	1.565 (1.100-2.227)^*^	1.549 (1.139-2.107)^**^
High diastolic blood pressure^k^	1.987 (1.346-2.934)^**^	1.894 (1.366-2.625)^**^
Metabolic Syndrome^l^	4.308 (2.130-8.727)^**^	4.789 (2.713-8.452)^**^

Data are expressed as prevalence ratio (95% confidence interval); MetS: metabolic syndrome; WC_UL_: waist circumference at the umbilical line; HDL-C: high density lipoprotein cholesterol.

^a^two smaller cutoff points suggested as of the best sensitivity and specificity relation (see Table 2).

^b^non-adjusted.

^c^adjusted for age, work position, physical activity level, smoker and alcohol consumption.

^d^glucose > 99 mg/dl.

^e^homeostatic model assessment insulin resistance (HOMA-IR) > 2.7.

^f^Total cholesterol > 200 mg/dl.

^g^HDL-C < 40 mg/dl.

^h^Triglycerides > 150 mg/d.

^i^Total cholesterol/HDL-C > 5.

^j^systolic blood pressure ≥ 140 mmHg.

^k^diastolic blood pressure ≥ 90 mmHg.

^l^Metabolic Syndrome prevalence by Alberti et al., [3].

^*^p ≤ 0.05; ^**^p ≤ 0.01; ^***^no data (null prevalence in one group).

## Discussion

This study was carried out to determine the anatomical point of waist circumference measurements that best identify overweight, obesity and central obesity, as measured by DXA, in Brazilian middle-aged men residents in the urban area of Viçosa city in the southeastern region of the country and to test the relationships of its cutoff points with metabolic syndrome, insulin resistance and other cardiometabolic risk factors.

We found that WC_UL_ exhibited a larger AUC than WC_MD_ and WC_NR_ and, therefore, it was more accurate in the identification of overweight and obesity making our results in line with the cutoff points recommended by SEEDO [26] and suggested by Bray et al. [27]. We also found that this circumference measure was strongly associated with %AAF (75th percentile), which makes of it also an accurate measure to identify central obesity. These results confirm the association of WC measurements with total and central adiposity as observed previously in different populations of similar ages. For example, similar findings were demonstrated in male urban residents in a city in the northeast of Brazil [19, 20] and also in men from other Latin American countries [18]. Likewise, these associations were found in Canadian (20–79 years old) [15] and Japanese (40–65 years old) [36] men. It is noteworthy that our data indicate the WC_UL_ as the best anatomical point to identify overweight, obesity and central obesity. This anthropometric indicator is of practical and clinical relevance in the professional field for both subject assessment and population-based studies. WC_UL_ has greater feasibility of location and measurement as well as lower error occurrence when it is measured, as compared to WC_NR_ and WC_MD_, especially in obese subjects.

Our results showed significant associations of the cutoff points for WC_UL_ (88.8 cm and 93.4 cm) with MetS, IR and other cardiometabolic risk factors assessed in this population. For instance, higher values for serum lipid profile, blood pressure and blood glucose, and a higher prevalence of MetS in subjects with WC_UL_ greater than 88.8 cm were observed. In addition, the prevalence of MetS, IR and cardiometabolic risk factors examined were even higher in subjects with WC_UL_ above 93.4 cm. Previous studies in different Brazilian geographical regions have pointed to a strong relationship between obesity, especially the central fat accumulation, and the occurrence of NCDs [37]. Thus, our data reinforce those previously reported for the Brazilian population and support the idea that the cutoff points obtained (88.8 cm and 93.4 cm) are of clinical and economic importance for risk assessment and prevention of obesity related diseases in this population, especially because Brazilian middle-aged men have high prevalence for mortality and morbidity related to NCDs [38].

Our findings represent a scenario in an apparently healthy middle-aged male population. The selected subjects who self-declared suffering from different diseases or disorders or taking medicines that could alter food intake and/or metabolism of nutrients were excluded from our study. In fact, the participants exhibited moderate alcohol consumption, low prevalence of smoking and were physically active. Despite that, the prevalence for overweight, dyslipidaemia, hypertriglyceridemia, high atherogenic index and hypertension was over 30%. Although these data reflect the reality of urban populations and alert for the danger of silent cardiometabolic diseases at this age, it cannot be extended to the national Brazilian population since there are regional ethnic groups and differences in eating habits across the country. In addition, it is worth to note that the suggested cutoff value for WC_UL_ cannot be generalized to all central circumferences measures as this may lead to possible errors of interpretation and misdiagnosis. In fact, in the present study population WC_UL_ was higher than both WC_NR_ and WC_MD_, which were not statistically different when compared to each other. Moreover, the increase of 19.3% in the central obesity prevalence using WC_UL_ ≥ 88.8 cm enables more accurate for cardiometabolic risk factors diagnosis.

Finally, our study presents some limitations: (a) despite the fact that the sample size was justified in relation to the target population, studies on larger populations including both gender residents in different regions of Brazil are needed to set cutoff points to the early diagnoses of NCDs nationwide; and (b) although the associations of the WC_UL_ cutoff point with MetS, IR and cardiometabolic risk factors remained after being adjusted for age, working position, physical activity level, smoking and alcohol consumption, other factors such as eating habits, marital status and family income were not included.

Overall, this cross-sectional study support the conclusions that: (a) the WC_UL_ measure seems to be the best anatomical point to perform waist circumference measurement to identify overweight, obesity and central obesity in urban Southeast Brazilian middle-aged men; and (b) the WC_UL_ cutoff point (88.8 cm) is significantly associated with MetS, IR and cardiometabolic risk factors in the studied population.

## Abbreviations

NCDs: Non-communicable diseases
MetS: Metabolic syndrome
IR: Insulin resistance
BMI: Body mass index
WC: Waist circumference
WC_NR_: Narrowest waist circumference
WC_MD_: Waist circumference measure in midpoint between the superior border of the iliac crest and inferior margin of the rib
WC_UL_: Waist circumference measure on umbilical line
DXA: Dual beam X-ray absorptiometry
%BF: Total body fat
%AAF: Abdominal area fat
SBP: Systolic blood pressure
DBP: Diastolic blood pressure
HDL-C: High-density lipoprotein
HOMA-IR: Homeostasis model assessment insulin resistance
AUC: Area under curve
ROC: Receiver operating characteristic curve
Sens: Sensitivity
Spec: Specificity.
